# Uptake of and Engagement With an Online Sexual Health Intervention (HOPE eIntervention) Among African American Young Adults: Mixed Methods Study

**DOI:** 10.2196/22203

**Published:** 2021-07-16

**Authors:** Alicia Williamson, Andrea Barbarin, Bettina Campbell, Terrance Campbell, Susan Franzen, Thomas M Reischl, Marc Zimmerman, Tiffany Christine Veinot

**Affiliations:** 1 School of Information University of Michigan Ann Arbor, MI United States; 2 IBM Watson Health Ann Arbor, MI United States; 3 YOUR Center Flint, MI United States; 4 TigerLIFE University of Memphis Memphis, TN United States; 5 Prevention Research Center of Michigan School of Public Health University of Michigan Ann Arbor, MI United States; 6 Department of Health Behavior and Health Education School of Public Health University of Michigan Ann Arbor, MI United States; 7 Department of Psychology University of Michigan Ann Arbor, MI United States

**Keywords:** HIV prevention, consumer health informatics, sexual health, health equity, technology adoption, technology usage

## Abstract

**Background:**

Regarding health technologies, African American young adults have low rates of uptake, ongoing usage, and engagement, which may widen sexual health inequalities.

**Objective:**

We aimed to examine rates of uptake and ongoing usage, and factors influencing uptake, ongoing usage, and engagement for a consumer health informatics (CHI) intervention for HIV/sexually transmitted infection (STI) prevention among African American young adults, using the diffusion of innovation theory, trust-centered design framework, and O’Brien and Toms’ model of engagement.

**Methods:**

This community-based participatory mixed methods study included surveys at four time points (n=315; 280 African American participants) among young adults aged 18 to 24 years involved in a blended offline/online HIV/STI prevention intervention (HIV Outreach, Prevention, and Education [HOPE] eIntervention), which was described as a “HOPE party.” Qualitative interviews were conducted with a subset of participants (n=19) after initial surveys and website server logs indicated low uptake and ongoing usage. A generalized linear mixed-effects model identified predictors of eIntervention uptake, server logs were summarized to describe use over time, and interview transcripts were coded and thematically analyzed to identify factors affecting uptake and engagement.

**Results:**

Participants’ initial self-reported eIntervention uptake was low, but increased significantly over time, although uptake never reached expectations. The most frequent activity was visiting the website. Demographic factors and HOPE party social network characteristics were not significantly correlated with uptake, although participant education and party network gender homophily approached significance. According to interviews, one factor driving uptake was the desire to share HIV/STI prevention information with others. Survey and interview results showed that technology access, perceived time, and institutional and technological trust were necessary conditions for uptake. Interviews revealed that factors undermining uptake were insufficient promotion and awareness building, and the platform of the intervention, with social media being less appealing due to previous negative experiences concerning discussion of sexuality on social media. During the interaction with the eIntervention, interview data showed that factors driving initial engagement were audience-targeted website esthetics and appealing visuals. Ongoing usage was impeded by insufficiently frequent updates. Similarly, lack of novelty drove disengagement, although a social media contest for sharing intervention content resulted in some re-engagement.

**Conclusions:**

To encourage uptake, CHI interventions for African American young adults can better leverage users’ desires to share information about HIV/STI prevention with others. Ensuring implementation through trusted organizations is also important, though vigorous promotion is needed. Visual appeal and targeted content foster engagement at first, but ongoing usage may require continual content changes. A thorough analysis of CHI intervention use can inform the development of future interventions to promote uptake and engagement. To guide future analyses, we present an expanded uptake and engagement model for CHI interventions targeting African American young adults based on our empirical results.

## Introduction

### Background

HIV and other sexually transmitted infections (STIs) are health concerns for African American young adults. In 2018, African Americans constituted 13% of the population but 42% of new HIV diagnoses in the United States [1], with young adults making up about 21% of new HIV diagnoses [2]. African American young adults are vulnerable to other STIs, having the highest rates of gonorrhea of all racial groups [3]. Consumer health informatics (CHI) technologies can support HIV/STI prevention by targeting outcomes such as sexual health literacy and HIV/STI testing rates [4].

However, previous experience with patient portals shows that CHI interventions may not achieve uptake and engagement among African Americans [5,6]. In an HIV/STI context, this may widen sexual health inequalities [7,8]. Numerous CHI interventions exist to prevent HIV [9-15] and STIs [16,17] among African American youth. Additionally, digital CHI interventions targeting African American populations can be effective at promoting safer sex behaviors [18,19] and can increase access to sexual health information [20,21]. However, few identify factors driving uptake and engagement [22]. While interventions have been effective at targeting African American youth [9,13,15], limited uptake and engagement can impede the promotion of behaviors, such as condom use [23,24], or the development of sexual health knowledge [25,26]. We define *uptake* as initiating contact with a CHI intervention, which is differentiated from *ongoing usage* of that intervention [27]. Uptake is rarely investigated, inconsistently defined, and often underreported [28,29]. *Engagement* has been defined by some as interactions over time [30], although information scientists use the term to demarcate the period in which a user interacts with an intervention [31]. Given our interest in experiences of CHI interventions, we use the latter definition of engagement in this study.

Factors driving uptake may explain disparities between racial groups in CHI intervention use [32]. Studies that examine uptake among African Americans suggest that it may be impeded by recruitment barriers, power differentials between researcher/clinician and participants, or researchers’ poor cultural competence [33]. Medical distrust stemming from discrimination and historical mistreatment also plays a role [34]. However, targeted recruitment and individual tailoring may improve uptake among African American young adults. Peer-driven recruitment [35,36] and partnering with community-based organizations [37] may increase uptake. Tailoring web-based interventions to users’ needs, interests, personalities, and contexts [38], and tailored messages from a personalized source [39] can also improve uptake.

CHI interventions should also foster ongoing usage. It can be difficult to differentiate between factors motivating uptake and ongoing usage; few studies have examined this. Some evidence shows that African American young adults’ engagement may be impeded by distrust, lack of time, limited technology access, and limited cultural relevance [32].

Some CHI interventions have successfully engaged African American users via social media, peer-led interventions, and designing to capture user attention. Social media-based HIV-prevention interventions have shown low attrition [14]. Peer-led CHI interventions have maintained engagement through existing social networks [14]. High levels of interest [40] and perceived usefulness may also drive ongoing usage [41].

Yet, there is limited understanding of what motivates uptake and engagement in African American young adults participating in CHI interventions to prevent HIV/STIs. We identify motivating factors for a tailored website and social media accounts promoting safer sex for African American young adults, framing our analyses with the theories described below.

### Theoretical Frameworks

#### Diffusion of Innovations

The diffusion of innovations (DOI) theory describes a process of communicating about innovations among members of social systems [42]. Communication can lead to adoption (deciding to use an intervention; this aligns with our definition of uptake). Personal characteristics, such as demographics, influence adoption. For example, people with more education and higher social participation tend to adopt innovations earlier. Furthermore, homophily (social similarity) with adopters increases the likelihood that nonadopters will make the subsequent decision to adopt an innovation. We used DOI to identify variables that may predict uptake.

Several HIV-prevention interventions have used the DOI theory [43]. Similarly, it informed the design of the HIV Outreach, Prevention, and Education (HOPE) eIntervention in this study. This intervention asked African American young adults to invite peers to HOPE parties (described below), which introduced the innovations (both the eIntervention and healthy sexual behaviors).

#### Trust-Centered Design Framework

**Figure 1:**
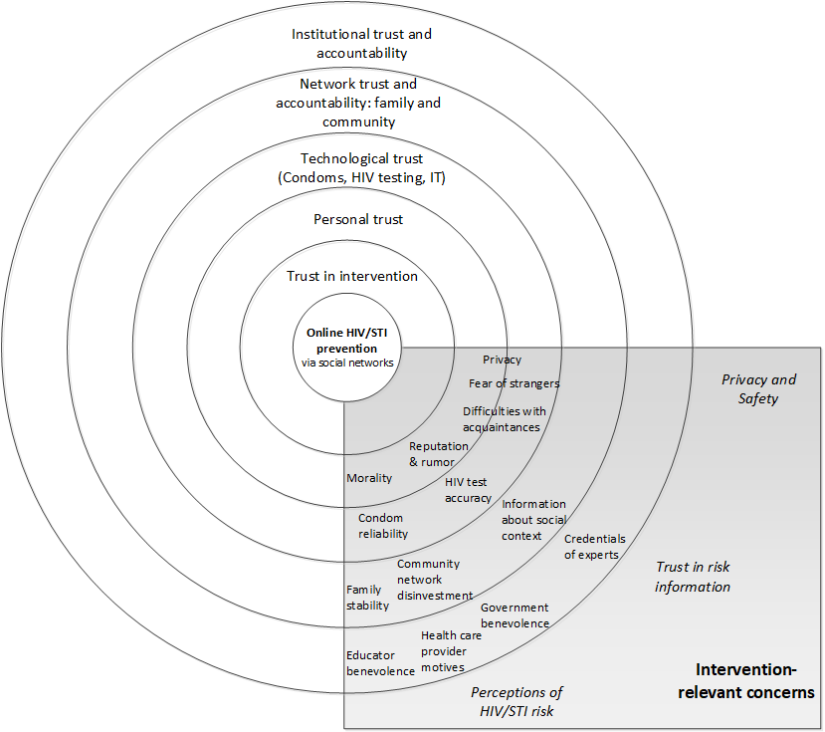
Trust-centered design framework. IT: information technology; STI: sexually transmitted infection.

The trust-centered design framework (Figure 1) [12] outlines trust-based needs in relation to CHI interventions at the personal, group, technological, and institutional levels for African American young adults. It centers trust in CHI interventions, calls for addressing trust-related requirements at multiple user-experience levels, and highlights forms of trust that may influence uptake and engagement. We used this framework to inform HOPE technology design and implementation strategies, and to analyze qualitative data in this study.

#### User Engagement Model

**Figure 2:**
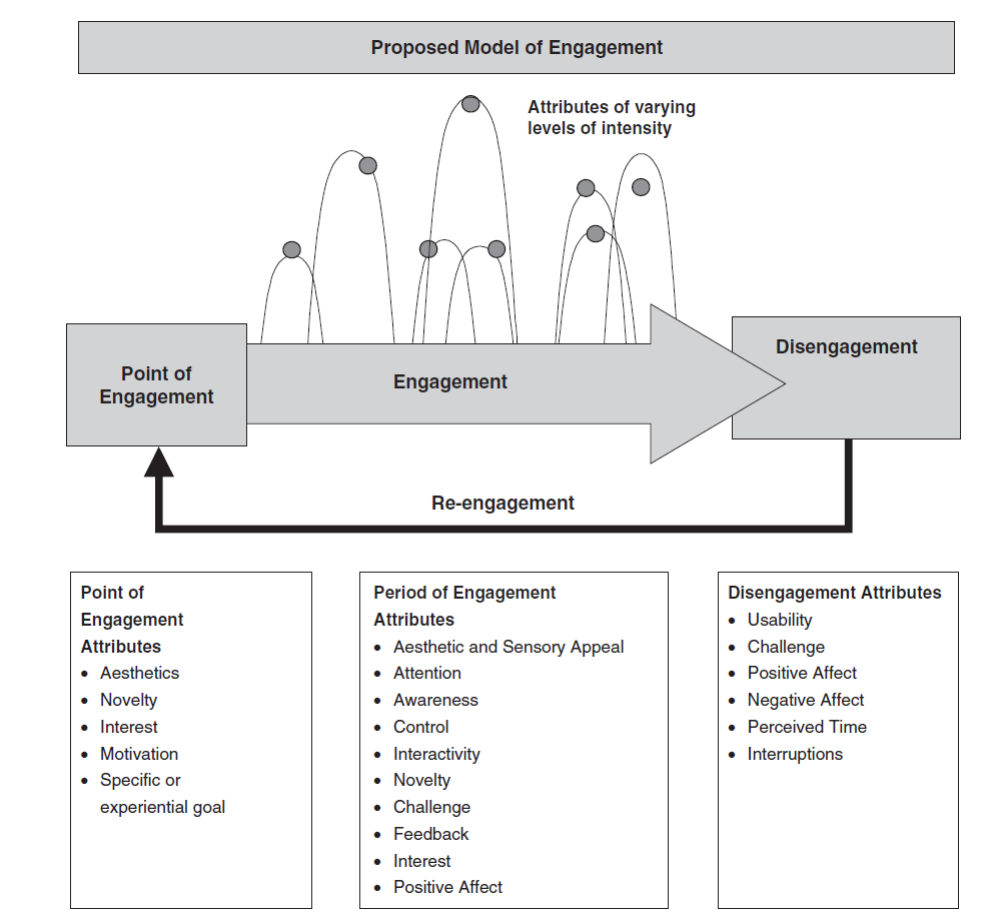
O’Brien and Toms’ model of engagement [31]. Reprinted with permission.

O’Brien and Toms’ model (Figure 2) focuses specifically on the period, or session, of interacting with an intervention. Focusing on websites as an intervention type, it differentiates the following four stages of engagement: point of engagement, period of engagement, disengagement, and re-engagement [31]. It posits that there are different drivers at each stage, grouped into (1) website features and (2) user experience features. Points of engagement and re-engagement are influenced by web features of esthetics and novelty, and user features including motivation. The period of engagement is influenced by sensory appeal, interactivity, novelty, challenge, feedback, attention, awareness, control, and positive affect. Disengagement is driven by website features of usability and challenge, and user features of positive/negative affect, perceived time, and interruptions. These factors vary in influence over time. We used this model to analyze qualitative interview data.

### Study Objectives

The aim of this study was to analyze uptake, ongoing usage, and engagement with a CHI intervention, the *HOPE eIntervention*, intended for African American young adults, which included a website and social media components. The research questions were as follows: RQ1: How much did HOPE participants use the eIntervention (uptake and ongoing usage)? RQ2: What factors were related to eIntervention uptake (initiating contact with the intervention)? RQ3: What factors were associated with ongoing usage and engagement while a participant interacted with the eIntervention?

## Methods

### Intervention Context

This mixed-methods, community-based participatory research (CBPR) [44] study included surveys and interviews with African American young adults who attended a community-based HIV/STI prevention intervention called a *HOPE party.* Regarding the CBPR approach, YOUR Center, a local faith-based nonprofit organization, was involved in study design, recruitment, implementation, data collection, and result dissemination, and the Genesee County Health Department assisted in recruitment; this is consistent with use of the CBPR approach in health informatics [44]. The team had monthly meetings during the intervention timeframe, and community members were involved in the design of the HOPE eIntervention, from its inception to its implementation. Furthermore, YOUR Center staff led the in-person interventions, and community members assisted by leveraging their social networks for recruitment. A community advisory board helped to integrate community needs throughout the project; this also aligns with CBPR practice [44]. Further information about the partnership can be found in a previous report [44].

### Intervention Description

HOPE parties were one-time, interactive, face-to-face HIV/STI prevention sessions hosted by African American young adults in their homes for their social networks. Attendees listened to didactic material, engaged in educational activities, and discussed safe sex practices with demonstrations of safe sex tools [41]. Local resources were also discussed, including information about the eIntervention. YOUR Center staff were trained to conduct the parties and used a facilitator’s manual to ensure that core content was covered in each party; fidelity assessments were also used at each party. However, as expected, there was some variation across parties. Recruitment was conducted at Genesee County STI clinics by trained UM School of Public Health, Health Professions and Studies students and community residents. YOUR Center staff also recruited participants through naturally occurring face-to-face professional and personal social networks, and snowball recruitment in which hosts recruited others.

The eIntervention, available to HOPE party participants, aimed to reinforce information presented at the parties and foster commitment to HIV/STI prevention. Together, the face-to-face parties and eIntervention comprised a blended offline/online intervention. The eIntervention was designed in collaboration with YOUR Center’s staff and community advisory board, and informed by design-oriented focus groups [12]. Based on the trust concerns when communicating about HIV/STIs that were raised in the trust-centered design framework developed as part of this study [12], the website allowed participants to remain anonymous to foster interactions that preserved privacy. Indeed, community partners expressed concerns about the acceptability of user accounts based on their interaction with the target audience for the intervention; thus, profiles were not required in order to access website content. Due to issues raised around inconsistent access to trusted information about sexual health [12,45], partnering with YOUR Center was intended to create an environment where African American young adult users would trust the information based on its institutional affiliation. Additionally, the blog features of the website allowed for users to access credible information via these short and casual posts. The website had a theme for mobile devices such as cellphones. A website plug-in detected what device was used to access the intervention and loaded either a desktop or mobile theme based on this detection. Users could toggle between the mobile and desktop versions of the website if desired.

At each HOPE party, YOUR Center staff and/or University of Michigan research staff introduced the eIntervention and distributed handouts that contained the website URL and directions on how to access social media accounts. The eIntervention consisted of a website with a blog, a Twitter account, and a Facebook page (examples of posts are included in Multimedia Appendix 1, Multimedia Appendix 2, and Multimedia Appendix 3). The website was updated at least once monthly. Facebook content included status updates, external links, photos, sexual health information, and/or questions. Tweets offered safer sex advice and memes reinforcing safer sex norms. Three social media contests incentivized eIntervention information sharing via social networks.

### Data Collection

HOPE party participants (n=315; 280 African American; some participants invited non-African American friends to parties) completed four surveys during the study. These included (1) at the party, in-person (baseline); (2) 3 months postparty, online/via phone (T1); (3) 6 months postparty, online/via phone (T2); and (4) 12 months postparty, online/via phone (T3). There were 57 HOPE parties over 3 years, with 1 to 12 participants at each who responded to surveys. Follow-up survey requests were first attempted over the phone, and those participants whom we could not reach were contacted via US mail and by email. We attempted to contact all survey participants regardless of the status of completion for the other follow-up waves. Participants received US $10 for completing the baseline, T1, and T2 questionnaires, and US $15 for completing the T3 questionnaire. Participants who completed all four surveys were entered into a draw for a US $100 gift card. The survey asked about demographic characteristics, social network information, and questions regarding use of the HOPE eIntervention. Surveys were developed to measure network assessments such as network composition (gender homophily) [46], social influence of networks based on density [47], and type of social tie (ie, weak or strong) [48] as predictors of HOPE website use. Survey questions used in the data analysis are detailed further in Multimedia Appendix 4.

After survey and website server logs indicated low uptake and engagement, individual in-depth semistructured interviews [49] (n=19) were conducted with African American young adult party participants. Interviews were conducted at least 1 year after each participant’s HOPE party. One graduate student (AB) from the University of Michigan who was trained in qualitative interviewing conducted the interviews using a semistructured interview guide that accommodated personalized follow-up questions. Using theoretical sampling [50], participants reporting low, medium, and high levels of eIntervention use on the survey were recruited via telephone or email. We contacted approximately 131 participants in total (56 participants with low levels of eIntervention use, 62 participants with medium levels of eIntervention use, and 12 participants with high levels of eIntervention use). Of the 131 participants, 19 participated in interviews (15% response rate).

We concluded interviews when we reached data saturation such that new data were no longer contributing new empirical insights [51]. Others have shown that data saturation can be achieved with as few as six to eight people in a relatively homogenous group such as that in this study (African American young adults in one geographic area) [52]. A timeline of the partnership with YOUR Center, development of the intervention, HOPE parties, follow-up surveys, and interviews is included in Multimedia Appendix 5.

Google Analytics measures included numbers of sessions, users, and page views; session duration; and trends. HOPE Twitter and Facebook data included numbers of followers/likes.

### Measures

#### Dependent Variable

At each survey wave, the main outcome in the individual-level statistical model for predicting uptake was a binary variable for having used the HOPE website or social media since the party. For the party-level model, this outcome was an average of the number of party attendees who used the eIntervention at each wave.

#### Independent Variables-Individual Level

##### Education

The DOI theory states that more education increases the speed of using innovations [42]; thus, educational level was a predictor. In the model, the variable had the following two levels: (1) less than high school diploma or General Education Diploma (GED) and (2) high school diploma or GED, some college, associate’s degree, or bachelor’s degree.

##### Gender

Gender was included as a binary predictor (male/female). This was included since men are more likely to have favorable technology attitudes [53], though these attitudes about technologies vary by age [54]. Further, women use health websites more [55] and are more likely to search for health information online [56].

##### Time Point

Participants’ use by time point was included because we observed increases in overall usage of the eIntervention over time.

#### Independent Variables-Party Level

##### Gender Homophily

The DOI theory predicts that homophily increases the odds of adoption, and our previous work has shown that gender homophily is associated with HIV testing behavior [46,57]. Thus, gender homophily was used as an independent variable, which was calculated using the Krackhardt E-I index network measure [58] as the number of females minus the number of males at the party divided by total participants [58].

##### Network Density

Network density [47] was included since it is linked to social influence [57]. This standard network measure [59] quantifies the number of participants at a party who have relationships divided by the number of total possible relationships between party members. Higher levels of network density are associated with health behavior transmission between youth [60,61]. This was calculated as the total number of attendee ties (any self-reported relationship before the party) divided by total possible ties [62].

##### Strong Tie Proportion

Strong ties [48] are also linked to social influence. They tell us the proportion of relationships between people at a party that are based on close interpersonal relationships [57]. Using a standard measure of tie strength, this survey question asked participants how close they felt to other party attendees, with the options being very close (strong ties), somewhat close, and not close [63]. There is ample evidence that people with whom one is emotionally intimate, such as close friends, influence one’s sexual risk behaviors [64-66]. This was measured by the number to whom each attendee felt very close divided by the number of existing ties between party members.

### Qualitative Interview Protocol

Interviews included (1) a demographic and technology use questionnaire, and (2) interviews about party experience, eIntervention uptake and engagement, internet and social media use, and HIV/STI-related communication practices.

### Data Analysis

#### Survey Analysis

Survey data were explored using descriptive statistics. Data analysis was conducted after the conclusion of T3. To identify uptake predictors, Stata was used to fit a generalized linear mixed-effects model [67] with binomial distribution to account for (1) the binary outcome variable (eIntervention use) and (2) repeated measures across the three time points. This method accounts for both within-participant and across-participant variability. The coefficients in this model can be interpreted as the log odds of predicted HOPE eIntervention use by variable. Due to our focus on African Americans, only the sample of African Americans (n=280) was entered into this model.

A second generalized linear mixed-effects model with a beta distribution using party-level indicators was fit. Uptake trends were also analyzed with respect to independent party-level variables such as gender homophily, network density, and strong tie proportion. There was a random effect for the person-level variable and were fixed effects for party ID (identifier) and within-party variables (gender homophily, strong tie proportion, and cohesion density) to predict average uptake at the party level while controlling for party size. For this model, the entire sample (n=315) was entered into the model as parties had both African American and non-African American attendees, although African Americans were the focus of the intervention design.

#### Interview Data Analysis

Interviews, averaging 43 minutes, were audio-recorded and transcribed. Among the 19 interview participants, 8 (42%) had low self-reported usage levels, 6 (32%) had medium self-reported usage levels, and 5 (26%) had high self-reported usage levels, with representation across usage levels. Transcripts were analyzed using deductive and inductive approaches in NVivo. Deductively, O’Brien and Toms’ [31] model of engagement and the trust-centered design framework [12] provided codes; line-by-line coding resulted in inductive codes. One coder initially coded the interviews, and a second coder reviewed the codes and reached consensus with the first. This resulted in emergent categories regarding information sharing, party experience, eIntervention experience, and social media [68]. Analytical memos were used to develop themes after reviewing and combining or collapsing codes [68]. Categories were also developed deductively using O’Brien and Toms’ model of engagement in the period, or session, in which users interacted with an intervention. Their model includes initial engagement, period of engagement, re-engagement, and disengagement. In this context, initial engagement is conceptualized as the beginning of the interaction with the eIntervention; period of engagement is the remaining time through which a participant uses the eIntervention; re-engagement is when a participant returns to engage with the eIntervention after days, weeks, or months; and disengagement is when a participant discontinues use of the eIntervention in a given session. Re-engagement and disengagement can be thought of as components of ongoing usage.

#### Website/Social Media Usage Data Analysis

Frequency counts were compiled for Google Analytics, Twitter, and Facebook data.

## Results

### Characteristics of the Participants

As Table 1 shows, 280 African American participants responded to the survey, and 19 African American young adult interviewees were drawn from this pool of respondents. Of the 280 survey respondents, 164 (58.6%) were female and 110 (39.3%) completed high school or equivalent, and the mean age was 21.13 years. Of the 19 interview participants, 14 (74%) were female, with similar education levels and a mean age of 24 years. As Table 2 shows, there were 57 parties in total, with an average of 5.63 (SD 2.56) participants per party who responded to the survey. Of the total 315 attendees who responded to the survey, 280 (88.9%) were African American.

**Table 1:** Summary of descriptive statistics for the African American sample at baseline.

Characteristic^a^		Quantitative survey (n=280)	Qualitative interview (n=19)
**Gender, n (%)**			
	Male	112 (40%)	5 (26%)
	Female	164 (59%)	14 (74%)
	Missing	4 (1%)	0 (0%)
Age at the time of the HOPE^b^ party, mean (SD)		21.13 (2.25)	24 (3.317)
**Education level at the time of the HOPE party, n (%)**			
	Less than high school diploma or GED^c^	80 (29%)	1 (5%)
	High school diploma or GED	110 (39%)	8 (42%)
	Some college, associate’s degree, or bachelor’s degree	85 (30%)	10 (53%)
	Missing	5 (2%)	0 (0%)
**Host, n (%)**			
	Yes	25 (9%)	3 (16%)
	No	255 (91%)	16 (84%)
	Missing	0 (0%)	0 (0%)
**Employment status^d^, n (%)**			
	Full-time work	30 (11%)	4 (21%)
	Part-time work	42 (15%)	1 (5%)
	Full-time student	34 (12%)	3 (16%)
	Part-time student	12 (4%)	2 (11%)
	Unemployed	165 (59%)	10 (53%)
	Other	14 (5%)	2 (11%)
	Missing	2 (0%)	0 (0%)

^a^All survey respondents identified as African American. Of the 19 qualitative interview respondents, 18 (95%) identified as African American and 1 (5%) identified as multiracial.

^b^HOPE: HIV Outreach, Prevention, and Education.

^c^GED: General Education Diploma.

^d^Participants could choose multiple employment statuses.

**Table 2:** Summary of descriptive statistics for baseline party characteristics.

Variable	Value
Total parties^a^, n (%)	57 (100)^b^
Total attendees per party^c^, mean (SD; min, max)	5.63 (2.56; 1, 12)
Average number of African American young adults per party^c^, mean (SD; min, max)	5 (2.16; 1, 11)
Average number of non-African American young adults per party^c^, mean (SD; min, max)	0.57 (1.22; 0, 6)
Average number missing per party^c^, mean (SD; min, max)	0.05 (0.23; 0, 1)
Average percentage of African American young adults per party^c^, mean (SD)	91.8% (16.7%)
Average percentage of non-African American young adults per party^c^, mean (SD)	7.4% (16.0%)
Average percentage missing per party^c^, mean (SD)	0.9% (3.8%)
Total attendees^c^, n (%)	315 (100%)
Total African American young adult attendees^c^, n (%)	280 (88.9%)
Total non-African American young adult attendees^c^, n (%)	32 (10.1%)
Total missing demographics^c^, n (%)	3 (1.0%)
Network density^c^, mean (SD; min, max)	0.80 (0.19; 0.4, 1.0)
Strong tie proportion^c^, mean (SD; min, max)	0.27 (0.17; 0.0, 0.8)
Party gender homophily^c^, mean (SD; min, max)	0.23 (0.58; −1.0, 1.0)

^a^Includes party participants who did not complete a survey.

^b^There were 57 total parties, but one party did not have any respondents fill out the survey at baseline, so 56 were included in the analyses.

^c^Includes only party participants who completed a survey.

### RQ1 Results

#### Survey Results

Initial uptake of the eIntervention and ongoing usage were low (Table 3). Self-reported use increased at subsequent time points. Initially, only 21 of 280 (8%) participants reported ever using the eIntervention (uptake), with an increase to 35 of 280 participants (13%) at T2 and 42 of 280 participants (15%) at T3. The most frequent engagements were visiting the website (T1: n=9, T2: n=24, T3: n=34), visiting the Facebook page (T1: n=1, T2: n=8, T3: n=11), and tweeting (T1: n=2, T2: n=4, T3: n=5). However, large amounts of survey data were missing either due to nonresponse at that time point or due to skipping that question (Table 3). Of the 359 total HOPE party participants, 315 participants took at least one survey. Regarding response at each time period, among 359 HOPE party participants, 178 responded at T1 (49.6% response rate), 180 responded at T2 (50.1% response rate), and 186 responded at T3 (51.8% response rate). Among the 280 African American party participants who ever answered the survey, 20 (7%) answered at only T1, 14 (5%) answered at only T2, and 16 (6%) answered at only T3. Table 3 outlines responses from the 280 African American respondents who ever responded to a survey.

**Table 3:** HIV Outreach, Prevention, and Education (HOPE) eIntervention use (N=280).

Variable			Time point 1, n (%)	Time point 2, n (%)	Time point 3, n (%)
**Use of the HOPE^a^ eIntervention since attending the HOPE party**					
	Yes		21 (7.5%)	35 (12.5%)	42 (15.0%)
	No		140 (50.0%)	127 (45.4%)	123 (43.9%)
	Missing^b^		119 (42.5%)	118 (42.1%)	115 (41.1%)
**What online HOPE activities did you do?^c^**					
	Visiting or using the HOPE website^d^		16 (76.2%)	24 (68.6%)	34 (80.1%)
	Visit the Facebook page		1 (4.8%)	8 (22.9%)	11 (26.2%)
	Post or blog on the HOPE website		0 (0%)	0 (0%)	1 (2.4%)
	Tweet on Twitter		2 (9.5%)	4 (11.4%)	5 (12.0%)
	Post on Facebook		0 (0%)	3 (8.6%)	1 (2.4%)
	Other		0 (0%)	3 (8.6%)	1 (2.4%)
**Why didn’t you use the HOPE eIntervention in the last 30 days?^c^**					
	No time		54 (41.9%)	60 (47.2%)	58 (46.0%)
	I did not have computer access		38 (29.5%)	31 (24.4%)	36 (28.6%)
	I did not want to		13 (10.1%)	23 (18.1%)	14 (11.1%)
	**Other**		24 (18.6%)	18 (14.2%)	17 (13.5%)
		I did not know about it^e^	10 (7.8%)	10 (7.9%)	7 (5.6%)
		I forgot about it^e^	8 (6.2%)	5 (3.9%)	6 (4.8%)

^a^HOPE: HIV Outreach, Prevention, and Education.

^b^Missing responses were either due to nonresponse at that time point or due to skipping the question.

^c^Participants were able to choose multiple responses.

^d^In the first time point (T1), participants were asked for their general use of the HOPE website as well as if they visited the website. In T2 and T3 surveys, the “general use” question was eliminated due to overlap.

^e^Open responses for “other” were categorized by “I did not know about it” and “I forgot about it.”

#### Log File and Social Media Results

According to the Google Analytics report, the website had 2432 sessions and 5754 total page views from July 2011 to March 2014, with an average session duration of 2 minutes 52 seconds. The Facebook page was updated with five to seven posts weekly and had 81 followers. HOPE Twitter was active from July 2011 to April 2014, with 131 followers. Inconsistency in reported usage may be due to missing survey responses or to eIntervention use by non-HOPE party participants.

### RQ2 Results

#### Survey Results

To identify uptake predictors across the three time points, we fitted a generalized linear mixed-effects model at the individual and party levels (Table 4). Individual-level results suggested that having some college education was a marginally significant predictor of eIntervention use. No other individual-level factors predicted uptake in our analysis of HOPE eIntervention use after the conclusion of T3 surveys, although there was an increase in usage from T1 to T2, as well as an increase from T1 to T3.

**Table 4:** Generalized linear mixed-effects model results.

Model		Coefficient	SE	Z	*P* > |z|	Confidence interval	
						Lower	Upper
**Individual-level model among African American party participants (n=280)**							
	Gender (female)	0.33	0.45	0.73	.47	−0.56	1.22
	Time point 1 to 2	0.89	0.37	2.40	.01	0.16	1.61
	Time point 2 to 3	1.12	0.37	3.05	.002	0.40	1.84
	High school diploma or GED^a^	0.59	0.48	1.22	.22	−0.36	1.54
	Some college, associate’s degree, or bachelor’s degree	0.93	0.48	1.92	.05	−0.02	1.88
	Network density	0.51	1.01	0.51	.61	−1.46	2.49
	Strong tie proportion	−0.53	1.25	−0.42	.67	−2.98	1.93
	Party gender homophily	0.995	0.72	1.38	.17	−0.42	2.41
**Party-level model among all party participants (n=315)**							
	Network density	−0.82	0.90	−0.92	.36	−2.58	0.93
	Strong tie proportion	1.46	0.98	1.49	.14	−0.46	3.37
	Gender homophily	0.05	0.03	1.81	.07	−0.004	0.11

^a^GED: General Education Diploma.

In the party-level model, gender homophily was the only marginally significant group-level variable, indicating a weak positive effect of a high proportion of females (gender homophily) on average uptake rates across participants in a given party.

As shown in Table 3, most participants who reported not having used the HOPE eIntervention in the past 30 days (of whom 100% had never used the intervention) at T1-T3 said this was due to lack of time (54-58 participants, 41.9%-47.2%). Following this, in frequency, were not having access to a computer (31-38 participants, 24.4%-29.5%), not wanting to (13-23 participants, 10.1%-18.1%), and other (17-24 participants, 13.5%-18.6%). Other responses concerned forgetting about the eIntervention or not being aware of it. Given their frequency, we interpreted perceived time and technology access as “necessary conditions” for uptake. Necessary conditions are facts that must be true for intervention uptake; however, they are insufficient on their own to promote uptake.

#### Interview Results

##### Individual Characteristics

###### Awareness

At least three interview participants were unaware of the eIntervention, although one mentioned visiting the website to see what was on it (Multimedia Appendix 6). Three participants suggested that uptake could be improved through more promotion.

###### Motivation

Some used the eIntervention with the goal of referring members of their community or family to it, believing they would benefit from learning about prevention (Multimedia Appendix 6). Furthermore, 12 interviewees shared information learned from the website with friends and family. Conversations with friends regarding HOPE usually involved sharing information about HIV/STI symptoms or testing locations (Multimedia Appendix 6).

##### Necessary Conditions

Necessary conditions were not typically enough to motivate uptake of the eIntervention, but their lack meant that people would not use it.

###### Technology Access

According to survey results, while some interview participants had internet access on varied devices, three did not have high-quality technology access, with two accessing it through others. Some participants also primarily accessed the internet on a mobile device; this limited their activities (Multimedia Appendix 6).

###### Perceived Time

Like survey respondents, some interviewees said they were too busy from work, school, and/or child care for the internet in general or for the HOPE eIntervention in particular (Multimedia Appendix 6).

###### Trust-Institutional

YOUR Center, which facilitated the parties, was considered a trustworthy information source (Multimedia Appendix 6). This perception was aided by personal connections. Three participants mentioned knowing someone linked to YOUR Center, and participants mentioned connections between HOPE parties and their churches. Attending parties organized and facilitated by individuals from trusted institutions made participants more likely to see the information from both the parties and eIntervention as credible.

###### Trust-Technological

Participants had trust in the website’s information, a form of technological trust [12,15]. Seven participants mentioned understanding the information easily, perceiving it as clear and having an appropriate length (Multimedia Appendix 6). However, participants mentioned negative experiences on social media that may have shaped their perceptions of discussing sexual health information online, potentially resulting in lower uptake of eIntervention social media components. People expressed concern about potential technology-facilitated privacy breaches and online fighting. Furthermore, gossip was a major concern. Ten discussed negative experiences with “Facebook Exposed” pages, a user-generated network of pages featuring incendiary posts disclosing personal information about sexual promiscuity, sexual identity, and HIV/STI status.

### RQ3 Results

#### Interview Results

##### Initial Engagement

###### Esthetics and Sensory Appeal

Participants responded positively to the website’s esthetics. Four liked its visual layout, stating it was not too simple and had multiple colors (Multimedia Appendix 6).

###### Challenge/Ease of Use

Participants valued that information on the website was direct, short, and engaging. Its content was easy to use. Compared to other websites about sexual health, the HOPE website was easier to use and thus more accessible (Multimedia Appendix 6).

##### Period of Engagement

###### Interactivity

The interactivity present on the website, such as the blog, was appreciated. However, three participants suggested making the website more interactive by imitating social media platforms. One suggested user profiles, much like social networking sites (Multimedia Appendix 6). This approach could also offer *novelty.*

##### Disengagement

###### Novelty

As Multimedia Appendix 6 shows, two participants who were initially regular website users stopped visiting due to a lack of consistent updates.

##### Re-engagement

###### Promotion of the Intervention Through Contests

Social media contests were intended to incentivize participants to visit the website to share information with peers; these fostered some limited re-engagement. Two interviewees participated in the contest, and one male participant said that he was interested in the financial reward and thought it was a good way to engage people with the content (Multimedia Appendix 6).

## Discussion

### Principal Results

This study identified factors promoting uptake and engagement with a CHI intervention for HIV/STI prevention among African American young adults. Uptake and ongoing usage were low overall; website uptake increased as more participants entered the study (Table 3), but always remained low. The only marginally significant individual-level positive predictor of uptake was education. There was also a marginally significant positive relationship between party gender homophily and party-level uptake. Awareness and motivation to share information with others positively influenced uptake. Necessary conditions undermined uptake when absent; these included technology access, perceived lack of time, and technological trust, especially regarding social media–based discussions about HIV/STIs. Visual appeal of the website, information with the appropriate level of challenge, and interactivity positively affected ongoing usage, although the website was not interactive enough for some participants. A social media contest also increased re-engagement (a component of ongoing usage), with limited reach. Lack of novelty was linked to disengagement (another component of ongoing usage).

### Comparison With Prior Work

**Figure 3:**
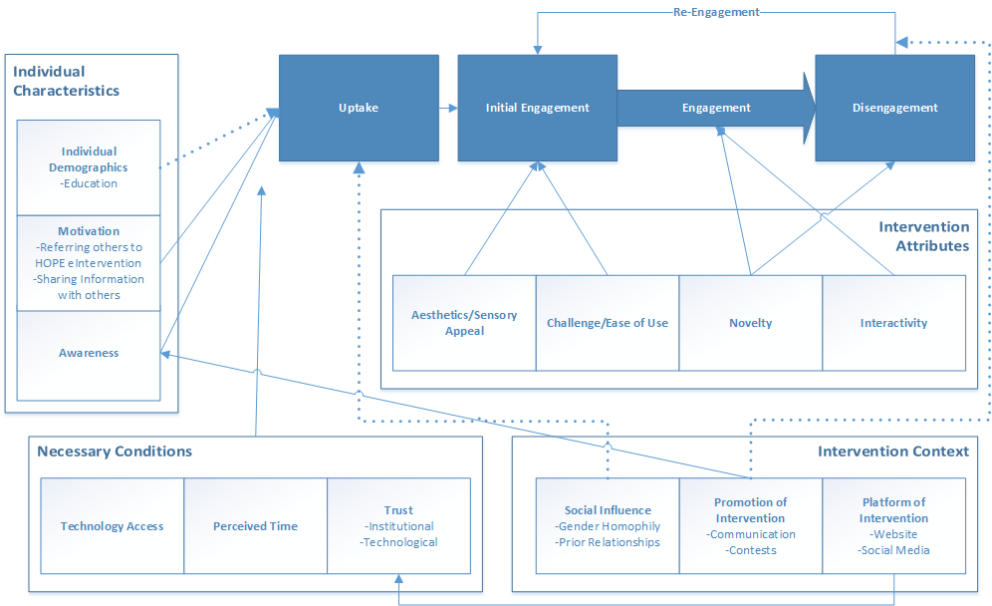
Uptake and engagement model for African American young adults. The dotted lines denote marginal significance. HOPE: HIV Outreach, Prevention, and Education.

Based on these findings, we extended O’Brien and Toms’ model of engagement [31] in a “Model of CHI Intervention Uptake and Engagement for African American Young Adults” (Figure 3). Our model reinforces O’Brien and Toms’ inclusion of motivation, challenge/ease of use, esthetics/sensory appeal, and novelty as factors influencing engagement; we found this in our interviews. To make the model more appropriate for this group, we added uptake. Factors driving uptake were identified through interviews (awareness and motivation) and survey data (education level and party network homophily). The model also newly incorporated necessary conditions and intervention context, including promotion efforts and the intervention’s platform on social media, which was linked to technological trust. Multimedia Appendix 7 presents which details support each element of the new model in Figure 3. With these additions, we offer interventionists a framework to identify culturally relevant factors for CHI design and implementation among African American young adults.

Of the three social influence variables studied, gender homophily weakly predicted uptake, aligning with the DOI theory. This comports with studies that found that network homophily influenced technology use [69,70] and increased adoption of new health behaviors in an online intervention [46]. Strong tie proportion and network density did not influence uptake, contrary to previous findings [47,48]. In prior work, strong ties and network density often resulted in the formation of subjective norms around technology use [48]. With the HOPE eIntervention, it is possible that no strong subjective norms existed. This could explain the insignificant results of the other social influence measures [71]. Future CHI intervention studies should promote normative behaviors regarding use to drive uptake.

Sharing HIV/STI information within social networks motivated uptake and engagement. This is consistent with the design of the parties as educational events within pre-existing social networks and our use of social media contests, as well as with prior work showing motivation to share health information within African American populations [72,73]. African American young adults often have limited access to HIV/STI information [45,74]. Sharing within social networks may help fill this gap, and this could be leveraged more effectively in future work. These results suggest that CHI interventions that target prevention may need to provide easily sharable information [75] applicable to the lives of the audience.

Trust in information sources is fundamental for African American technology users [12,76,77]. HOPE users’ trust in YOUR Center had an effect on uptake of the HOPE parties, which in turn affected eIntervention uptake. The website incorporated design elements aligned with recommendations posited by a meta-analysis [78] to influence trust, such as lack of login, in order to protect privacy [74]. CHI interventions for this population must incorporate trust-related considerations, including privacy and security [79].

While the website was designed to protect privacy [12], uptake may have been affected by participants’ prior experiences with the discussion of HIV/STI information online, and other trust-related concerns observed in prior work. Observing negative interactions shapes how individuals engage with online interventions [80] and manage online identities [81]. Therefore, these experiences might have particularly deterred use of interactive intervention elements, such as commenting on the blog. Furthermore, interactions, such as *liking* a Facebook page or *following* a Twitter account, may have been identifying [82] enough to discourage doing so. While African American young adults may believe that social media can helpfully disseminate health information [83], HIV/STI information may be too sensitive to discuss publicly.

Participants expressed interest in increased interactivity and similarity to sites such as Facebook. Modeling interventions after social media sites may promote engagement. Young adults have the highest social media usage among age groups [84] and may expect interventions to mirror their interactivity. Notably, although a blog provided interactivity on the website, decreasing use of blogs among youth over time [85] indicates that blogs may not have had the requisite popularity with the audience to provide them with desired interactivity. Additionally, given the low uptake of social media–based HOPE accounts, websites targeting HIV prevention or other health issues for youth could implement other components, such as newsfeeds, thus allowing for passive engagement [86,87].

The website fostered initial engagement with esthetically pleasing visuals and audience-appropriate information. Webpage attractiveness may be as important as content for engaging African American young adults [17], who prefer attention-getting colors [88]. Perceived information quality and ease of use also drove engagement. CHI interventions targeting specific populations should present content that is culturally relevant, accessible, and perceived as trustworthy, such as through links to social networks and trusted institutions [75].

Others have shown that lack of time and technology access may be uptake barriers [29,37]. Many survey respondents said that they lacked time to use the eIntervention. More advantaged participants are more likely to have time to engage with health promotion efforts [89], so young African Americans may particularly need CHI designs with low time burden. Additionally, a significant minority of party participants said they did not use the eIntervention because they lacked technology access. African Americans are less likely than white individuals to own computers or have broadband and are more likely to be “smartphone only” internet users [90]. Interventions targeting African American young adults or other groups that may not have access to consistent home broadband should be optimized for cellphones.

Despite attempts to increase uptake and re-engagement through face-to-face introductions to the eIntervention at HOPE parties and social media contests with financial incentives, low uptake may have resulted from insufficient promotion or limited usefulness [91]. Research on African American health intervention use found that face-to-face interactions have a greater impact on health outcomes [92]. Including more in-person promotional campaigns before implementing an intervention may be helpful.

### Limitations

This study has some limitations. African American young adults were sampled from one county in one state. While we cannot generalize the results to broader populations, they could be extended to groups at a high risk for acquiring HIV/STIs in other urban settings in the United States. The survey and interview relied on participant self-report, which may have desirability effects; however, this mixed-methods approach allowed for diverse data to inform our analysis. Large amounts of data were missing in the self-report questionnaires, largely due to difficulties with follow-up despite attempts using listed phone numbers and mailing addresses, which is a common obstacle in community-engaged research. Additionally, while Google Analytics, Facebook, and Twitter data provided descriptive information about website use, we could not ensure that individual visitors were party participants because we did not implement user accounts due to privacy concerns and the website was accessible via public search [12]. Additionally, many of the respondents reported being unemployed at the point of the baseline survey, but they may have begun employment or schooling during the follow-up period.

### Conclusion

Our study identified factors driving uptake and engagement within an HIV-prevention CHI intervention with African American young adults. We affirm and extend O’Brien and Toms’ model to include uptake, individual factors, necessary conditions, and context as displayed in Figure 3. Findings revealed that CHI interventions for prevention among African American young adults should facilitate peer information sharing, be implemented through trusted organizations, be interactive, and offer attention-grabbing esthetic designs, accessible information, and regular updates. Intervention design and implementation must also foster trust and address barriers such as lack of time.

## Appendix

Multimedia Appendix 1 HIV Outreach, Prevention, and Education (HOPE) eIntervention components: website.

Multimedia Appendix 2 HIV Outreach, Prevention, and Education (HOPE) eIntervention components: Twitter.

Multimedia Appendix 3 HIV Outreach, Prevention, and Education (HOPE) eIntervention components: Facebook post.

Multimedia Appendix 4 Survey instrument.

Multimedia Appendix 5 Timeline of community partnership, intervention development, HOPE parties, and data collection. HOPE: HIV Outreach, Prevention, and Education.

Multimedia Appendix 6 Extended model of user engagement based on qualitative interview data.

Multimedia Appendix 7 Support for each element of the proposed model.

## Abbreviations

CBPR: community-based participatory research
CHI: consumer health informatics
DOI: diffusion of innovations
GED: General Education Diploma
HOPE: HIV Outreach, Prevention, and Education
STI: sexually transmitted infection

## References

[1] HIV and African American People. Centers for Disease Control and Prevention.

[2] (2021). HIV and Youth. Centers for Disease Control and Prevention.

[3] (2018). Sexually Transmitted Disease Surveillance 2017. Centers for Disease Control and Prevention.

[4] LeGrand S, Muessig KE, Horvath KJ, Rosengren AL, Hightow-Weidman LB (2017). Using technology to support HIV self-testing among MSM. Curr Opin HIV AIDS.

[5] Goel MS, Brown TL, Williams A, Hasnain-Wynia R, Thompson JA, Baker DW (2011). Disparities in enrollment and use of an electronic patient portal. J Gen Intern Med.

[6] Lyles C, Harris L, Jordan L, Grothaus L, Wehnes L, Reid R, Ralston JD (2012). Patient race/ethnicity and shared medical record use among diabetes patients. Med Care.

[7] Ragsdale A, Rotheram-Borus M (2015). Re-shaping HIV Interventions with Technology. AIDS Behav.

[8] Veinot TC, Mitchell H, Ancker JS (2018). Good intentions are not enough: how informatics interventions can worsen inequality. J Am Med Inform Assoc.

[9] Danielson C, McCauley J, Gros K, Jones A, Barr S, Borkman A, Bryant BG, Ruggiero KJ (2016). SiHLEWeb.com: Development and usability testing of an evidence-based HIV prevention website for female African-American adolescents. Health Informatics J.

[10] Holloway IW, Winder TJ, Lea CH, Tan D, Boyd D, Novak D (2017). Technology Use and Preferences for Mobile Phone-Based HIV Prevention and Treatment Among Black Young Men Who Have Sex With Men: Exploratory Research. JMIR Mhealth Uhealth.

[11] Patel VV, Masyukova M, Sutton D, Horvath KJ (2016). Social Media Use and HIV-Related Risk Behaviors in Young Black and Latino Gay and Bi Men and Transgender Individuals in New York City: Implications for Online Interventions. J Urban Health.

[12] Veinot TC, Campbell TR, Kruger DJ, Grodzinski A (2013). A question of trust: user-centered design requirements for an informatics intervention to promote the sexual health of African-American youth. J Am Med Inform Assoc.

[13] Voisin DR, Tan K, DiClemente RJ (2013). A longitudinal examination of sexually transmitted infection/HIV prevention knowledge and sexually transmitted infections among African-American adolescent females. J Health Psychol.

[14] Young LE, Schumm P, Alon L, Bouris A, Ferreira M, Hill B, Khanna AS, Valente TW, Schneider JA (2018). PrEP Chicago: A randomized controlled peer change agent intervention to promote the adoption of pre-exposure prophylaxis for HIV prevention among young Black men who have sex with men. Clin Trials.

[15] Voisin DR, Tan K, Salazar LF, Crosby R, DiClemente RJ (2012). Correlates of sexually transmitted infection prevention knowledge among African American girls. J Adolesc Health.

[16] DiClemente R, Davis T, Swartzendruber A, Fasula A, Boyce L, Gelaude D, Gray SC, Hardin J, Rose E, Carry M, Sales JM, Brown JL, Staples-Horne M (2014). Efficacy of an HIV/STI sexual risk-reduction intervention for African American adolescent girls in juvenile detention centers: a randomized controlled trial. Women Health.

[17] Downs J, Murray P, Bruine de Bruin W, Penrose J, Palmgren C, Fischhoff B (2004). Interactive video behavioral intervention to reduce adolescent females' STD risk: a randomized controlled trial. Soc Sci Med.

[18] Bond K, Ramos S (2019). Utilization of an Animated Electronic Health Video to Increase Knowledge of Post- and Pre-Exposure Prophylaxis for HIV Among African American Women: Nationwide Cross-Sectional Survey. JMIR Form Res.

[19] Wingood GM, Card JJ, Er D, Solomon J, Braxton N, Lang D, Seth P, Cartreine J, Diclemente RJ (2011). Preliminary efficacy of a computer-based HIV intervention for African-American women. Psychol Health.

[20] Kalichman SC, Cherry C, Cain D, Pope H, Kalichman M, Eaton L, Weinhardt L, Benotsch EG (2006). Internet-based health information consumer skills intervention for people living with HIV/AIDS. J Consult Clin Psychol.

[21] Teadt S, Burns J, Montgomery T, Darbes L (2020). African American Adolescents and Young Adults, New Media, and Sexual Health: Scoping Review. JMIR Mhealth Uhealth.

[22] Skovdal M (2019). Facilitating engagement with PrEP and other HIV prevention technologies through practice-based combination prevention. J Int AIDS Soc.

[23] St Lawrence J, Eldridge G, Reitman D, Little C, Shelby M, Brasfield T (1998). Factors influencing condom use among African American women: implications for risk reduction interventions. Am J Community Psychol.

[24] Young SD, Holloway I, Jaganath D, Rice E, Westmoreland D, Coates T (2014). Project HOPE: online social network changes in an HIV prevention randomized controlled trial for African American and Latino men who have sex with men. Am J Public Health.

[25] St Lawrence J, Brasfield T, Jefferson K, Alleyne E, O'Bannon R, Shirley A (1995). Cognitive-behavioral intervention to reduce African American adolescents' risk for HIV infection. J Consult Clin Psychol.

[26] Wadham E, Green C, Debattista J, Somerset S, Sav A (2019). New digital media interventions for sexual health promotion among young people: a systematic review. Sex Health.

[27] Emery J, Coleman T, Sutton S, Cooper S, Leonardi-Bee J, Jones M, Naughton F (2018). Uptake of Tailored Text Message Smoking Cessation Support in Pregnancy When Advertised on the Internet (MiQuit): Observational Study. J Med Internet Res.

[28] Donkin L, Christensen H, Naismith SL, Neal B, Hickie IB, Glozier N (2011). A systematic review of the impact of adherence on the effectiveness of e-therapies. J Med Internet Res.

[29] Price M, Gros DF, McCauley JL, Gros KS, Ruggiero KJ (2012). Nonuse and dropout attrition for a web-based mental health intervention delivered in a post-disaster context. Psychiatry.

[30] Alkhaldi G, Hamilton F, Lau R, Webster R, Michie S, Murray E (2016). The Effectiveness of Prompts to Promote Engagement With Digital Interventions: A Systematic Review. J Med Internet Res.

[31] O'Brien H, Toms E (2008). What is user engagement? A conceptual framework for defining user engagement with technology. J. Am. Soc. Inf. Sci.

[32] Christopher Gibbons M (2011). Use of health information technology among racial and ethnic underserved communities. Perspect Health Inf Manag.

[33] Dancy BL, Wilbur J, Talashek M, Bonner G, Barnes-Boyd C (2004). Community-based research: barriers to recruitment of African Americans. Nurs Outlook.

[34] Oh A, Chou W, Jackson D, Cykert S, Jones N, Schaal J, Hesse BW, Ahern DK, Beckjord E (2016). Chapter 2 - Reducing Cancer Disparities Through Community Engagement: The Promise of Informatics. Oncology Informatics: Using Health Information Technology to Improve Processes and Outcomes in Cancer.

[35] Broadhead RS, Heckathorn DD, Weakliem DL, Anthony DL, Madray H, Mills RJ, Hughes J (1998). Harnessing peer networks as an instrument for AIDS prevention: results from a peer-driven intervention. Public Health Rep.

[36] Latkin CA, Sherman S, Knowlton A (2003). HIV prevention among drug users: outcome of a network-oriented peer outreach intervention. Health Psychol.

[37] Danielson CK, McCauley JL, Jones AM, Borkman AL, Miller S, Ruggiero KJ (2013). Feasibility of delivering evidence-based HIV/STI prevention programming to a community sample of African American teen girls via the internet. AIDS Educ Prev.

[38] Kelders SM (2012). Understanding adherence to web-based interventions. University of Twente.

[39] Strecher VJ, McClure J, Alexander G, Chakraborty B, Nair V, Konkel J, Greene S, Couper M, Carlier C, Wiese C, Little R, Pomerleau C, Pomerleau O (2008). The role of engagement in a tailored web-based smoking cessation program: randomized controlled trial. J Med Internet Res.

[40] Hightow-Weidman LB, Pike E, Fowler B, Matthews DM, Kibe J, McCoy R, Adimora AA (2012). HealthMpowerment.org: feasibility and acceptability of delivering an internet intervention to young Black men who have sex with men. AIDS Care.

[41] Marsch LA, Grabinski MJ, Bickel WK, Desrosiers A, Guarino H, Muehlbach B, Solhkhah R, Taufique S, Acosta M (2011). Computer-assisted HIV prevention for youth with substance use disorders. Subst Use Misuse.

[42] Rogers EM (2003). Diffusion of Innovations. Fifth Edition.

[43] Singhal A, Rogers E (2003). Combating AIDS: Communication strategies in action.

[44] Unertl K, Schaefbauer C, Campbell T, Senteio C, Siek K, Bakken S, Veinot TC (2016). Integrating community-based participatory research and informatics approaches to improve the engagement and health of underserved populations. J Am Med Inform Assoc.

[45] Kimmel A, Williams T, Veinot T, Campbell B, Campbell T, Valacak M, Kruger DJ (2013). 'I make sure I am safe and I make sure I have myself in every way possible': African-American youth perspectives on sexuality education. Sex Educ.

[46] Centola D (2011). An experimental study of homophily in the adoption of health behavior. Science.

[47] Haythornthwaite C (2016). Online Personal Networks. New Media & Society.

[48] ten Kate S, Haverkamp S, Mahmood F, Feldberg F (2010). Social network influences on technology acceptance: a matter of tie strength, centrality and density. BLED 2010 Proceeding.

[49] Gubrium J, Holstein J, Marvasti A, McKinney K (2012). The SAGE Handbook of Interview Research: The Complexity of the Craft.

[50] Glaser B, Strauss A, Denzin NK (2017). Theoretical Sampling. Sociological Methods.

[51] Saunders B, Sim J, Kingstone T, Baker S, Waterfield J, Bartlam B, Burroughs H, Jinks C (2018). Saturation in qualitative research: exploring its conceptualization and operationalization. Qual Quant.

[52] Guest G, Bunce A, Johnson L (2016). How Many Interviews Are Enough?. Field Methods.

[53] Cai Z, Fan X, Du J (2017). Gender and attitudes toward technology use: A meta-analysis. Computers & Education.

[54] James DCS, Harville C (2017). Barriers and Motivators to Participating in mHealth Research Among African American Men. Am J Mens Health.

[55] Cline RJ, Haynes KM (2001). Consumer health information seeking on the Internet: the state of the art. Health Educ Res.

[56] Wimble M (2016). Understanding Health and Health-Related Behavior of Users of Internet Health Information. Telemed J E Health.

[57] Veinot TC, Caldwell E, Loveluck J, Arnold MP, Bauermeister J (2016). HIV Testing Behavior and Social Network Characteristics and Functions Among Young Men Who have Sex with Men (YMSM) in Metropolitan Detroit. AIDS Behav.

[58] Krackhardt D, Stern RN (1988). Informal Networks and Organizational Crises: An Experimental Simulation. Social Psychology Quarterly.

[59] Wasserman S, Faust K (1994). Social Network Analysis: Methods and Applications.

[60] Cook SH, Bauermeister JA, Gordon-Messer D, Zimmerman MA (2013). Online network influences on emerging adults' alcohol and drug use. J Youth Adolesc.

[61] Haynie DL (2001). Delinquent Peers Revisited: Does Network Structure Matter?. American Journal of Sociology.

[62] Smith AMA, Grierson J, Wain D, Pitts M, Pattison P (2004). Associations between the sexual behaviour of men who have sex with men and the structure and composition of their social networks. Sex Transm Infect.

[63] Boase J, Horrigan JB, Wellman B, Rainie L (2006). The strength of internet ties.

[64] Billy JO, Udry JR (1985). The influence of male and female best friends on adolescent sexual behavior. Adolescence.

[65] Choi K, Ayala G, Paul J, Boylan R, Gregorich S (2013). Social network characteristics and HIV risk among African American, Asian/Pacific Islander, and Latino men who have sex with men. J Acquir Immune Defic Syndr.

[66] Walter HJ, Vaughan RD, Gladis MM, Ragin DF, Kasen S, Cohall AT (1992). Factors associated with AIDS risk behaviors among high school students in an AIDS epicenter. Am J Public Health.

[67] McCulloch C, Neuhaus J (2005). Generalized Linear Mixed Models. Encyclopedia of Biostatistics.

[68] Saldaña J (2015). The Coding Manual for Qualitative Researchers.

[69] Aral S, Muchnik L, Sundararajan A (2009). Distinguishing influence-based contagion from homophily-driven diffusion in dynamic networks. Proc Natl Acad Sci U S A.

[70] Park SY (2009). An analysis of the technology acceptance model in understanding university students' behavioral intention to use e-learning. Educational Technology & Society.

[71] Schepers J, Wetzels M (2007). A meta-analysis of the technology acceptance model: Investigating subjective norm and moderation effects. Information & Management.

[72] Jones LM, Wright KD, Wallace MK, Veinot TC (2018). "Take an opportunity whenever you get it": Information Sharing among African-American Women with Hypertension. J Assoc Inf Sci Technol.

[73] Veinot TC (2009). Interactive acquisition and sharing: Understanding the dynamics of HIV/AIDS information networks. J. Am. Soc. Inf. Sci.

[74] Guilamo-Ramos V, Lee JJ, Kantor LM, Levine DS, Baum S, Johnsen J (2015). Potential for using online and mobile education with parents and adolescents to impact sexual and reproductive health. Prev Sci.

[75] Bauermeister JA, Pingel ES, Jadwin-Cakmak L, Harper GW, Horvath K, Weiss G, Dittus P (2015). Acceptability and preliminary efficacy of a tailored online HIV/STI testing intervention for young men who have sex with men: the Get Connected! program. AIDS Behav.

[76] Paige SR, Krieger JL, Stellefson ML (2017). The Influence of eHealth Literacy on Perceived Trust in Online Health Communication Channels and Sources. J Health Commun.

[77] Richardson A, Allen JA, Xiao H, Vallone D (2012). Effects of race/ethnicity and socioeconomic status on health information-seeking, confidence, and trust. J Health Care Poor Underserved.

[78] Vega LC, Montague E, Dehart T (2011). Trust between patients and health websites: a review of the literature and derived outcomes from empirical studies. Health Technol (Berl).

[79] Gibbons MC, Fleisher L, Slamon RE, Bass S, Kandadai V, Beck JR (2011). Exploring the potential of Web 2.0 to address health disparities. J Health Commun.

[80] Veinot TC, Campbell T, Kruger D, Grodzinski A, Franzen S (2011). Drama and danger: the opportunities and challenges of promoting youth sexual health through online social networks. AMIA Annu Symp Proc.

[81] Evers C, Albury K, Byron P, Crawford K (2013). Young people, social media, social network sites and sexual health communication in Australia: "this is funny, you should watch it". International Journal of Communication.

[82] Dwyer C (2011). Privacy in the Age of Google and Facebook. IEEE Technol. Soc. Mag.

[83] Klein H, Sterk CE, Elifson KW (2016). Knowledge about HIV in a Community Sample of Urban African Americans in the South. J AIDS Clin Res.

[84] (2014). Social Media Fact Sheet. Pew Research Center.

[85] (2010). Social Media and Young Adults. Pew Research Center.

[86] Bayer JB, Triệu P, Ellison NB (2020). Social Media Elements, Ecologies, and Effects. Annu Rev Psychol.

[87] Burke M, Kraut R, Marlow C (2011). Social capital on Facebook: differentiating uses and users. CHI '11: Proceedings of the SIGCHI Conference on Human Factors in Computing Systems.

[88] Lazard AJ, Pikowski J, Horrell L, Ross JC, Noar SM, Sutfin EL (2020). Adolescents' and Young Adults' Aesthetics and Functionality Preferences for Online Tobacco Education. J Cancer Educ.

[89] Phelan JC, Link BG (2015). Is Racism a Fundamental Cause of Inequalities in Health?. Annu. Rev. Sociol.

[90] (2019). Smartphones help Blacks, Hispanics bridge some – but not all – digital gaps with whites. Pew Research Center.

[91] Anhøj J, Jensen AH (2004). Using the internet for life style changes in diet and physical activity: a feasibility study. J Med Internet Res.

[92] Montague E, Perchonok J (2012). Health and wellness technology use by historically underserved health consumers: systematic review. J Med Internet Res.

