# Supplementary figures and images for: Uptake of and Engagement With an Online Sexual Health Intervention (HOPE eIntervention) Among African American Young Adults: Mixed Methods Study
Source: J Med Internet Res. 2021 Jul 16;23(7):e22203. doi: 10.2196/22203 (PMC8325088; doi:10.2196/22203)

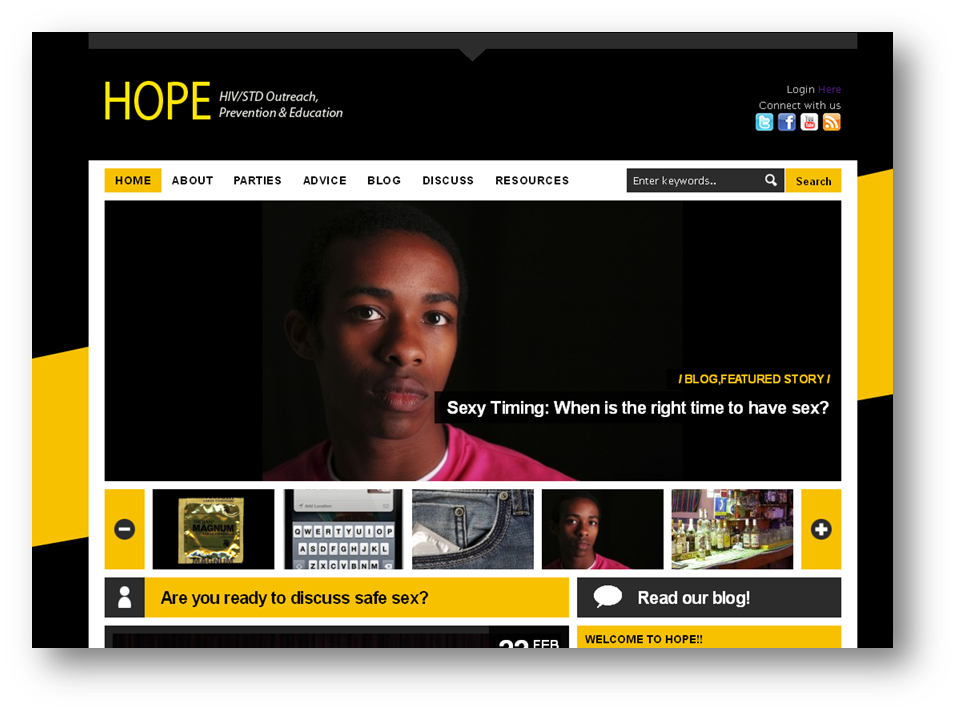

Supplement: Multimedia Appendix 1 [file jmir_v23i7e22203_app1.png]

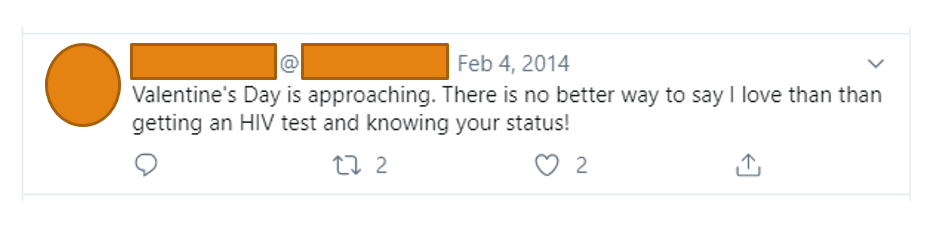

Supplement: Multimedia Appendix 2 [file jmir_v23i7e22203_app2.png]

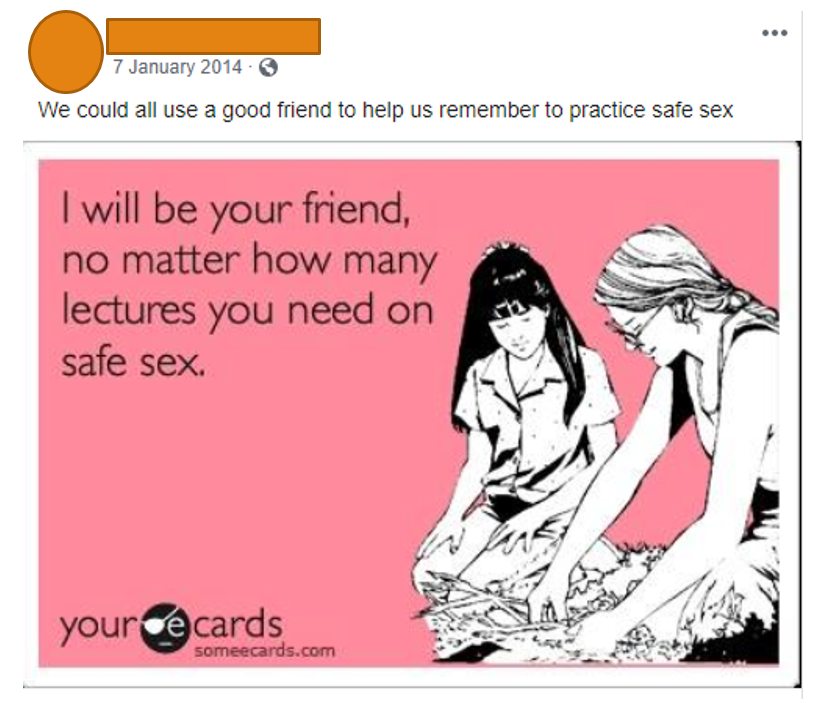

Supplement: Multimedia Appendix 3 [file jmir_v23i7e22203_app3.png]

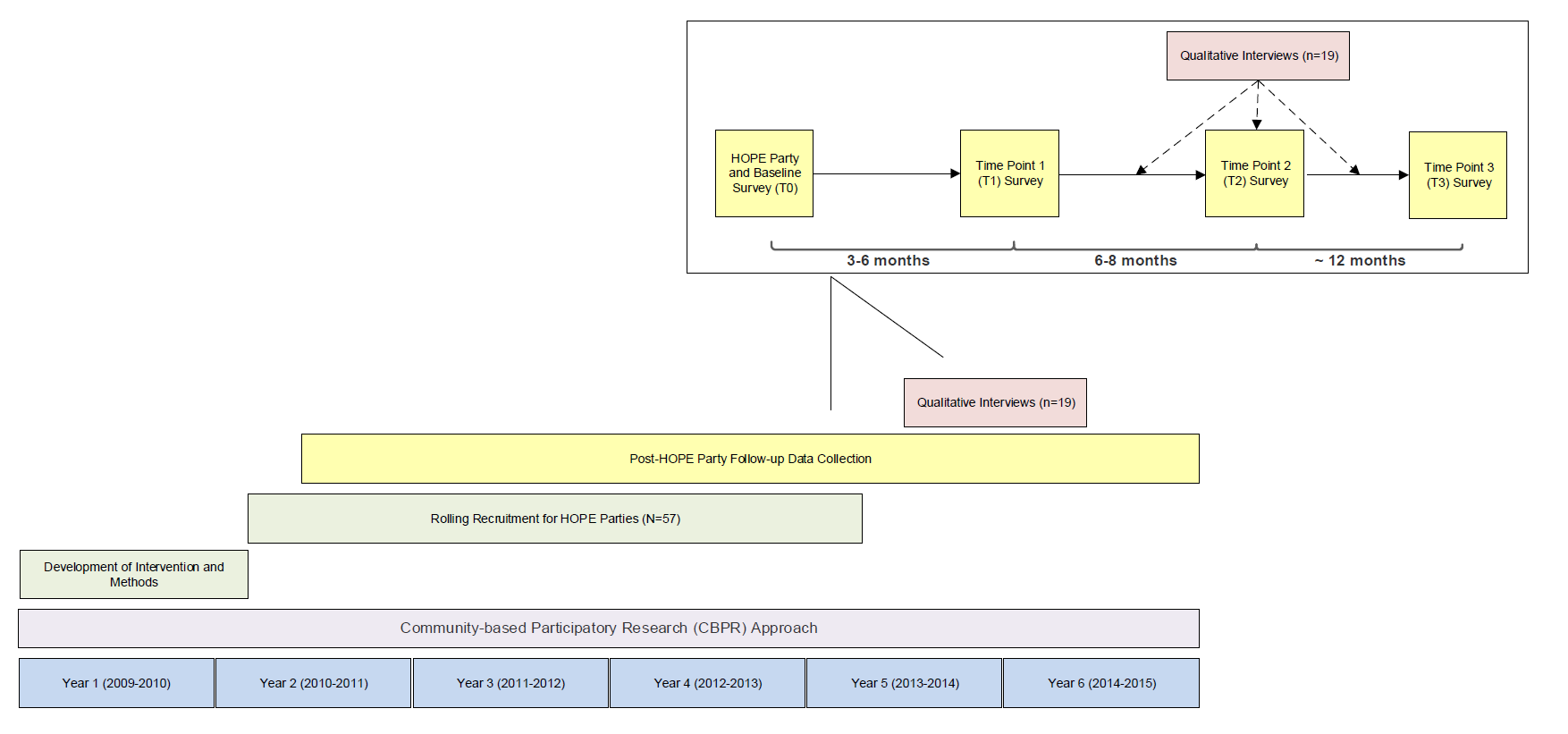

Supplement: Multimedia Appendix 5 [file jmir_v23i7e22203_app5.png]
