# Supplementary material for: Uptake of and Engagement With an Online Sexual Health Intervention (HOPE eIntervention) Among African American Young Adults: Mixed Methods Study
Source: J Med Internet Res. 2021 Jul 16;23(7):e22203. doi: 10.2196/22203 (PMC8325088; doi:10.2196/22203)
Supplement: Multimedia Appendix 4 [file jmir_v23i7e22203_app4.doc]

Appendix 4. Survey Instruments

**BASELINE QUESTIONS**

**What is your gender?** *(Please check one.)*

- Male
- Female

**What is your Birth Month? ___________ What is your Birth Year? _________**

**What is your race?** *(Check all that apply.)*

- Black or African American
- White
- American Indian or Alaskan Native
- Asian
- Native Hawaiian or Pacific Islander
- Other: _______________________

**Are you Spanish or Hispanic or Latino?** *(Please check one.)*

- Yes
- No

**What is the highest degree or level of school that you have completed?**

*(Please check one.)*

- Grade 8 or less
- Grades 9 to 12, no diploma
- High school graduate or equivalent (GED)
- Some college
- Associate degree (e.g., AA, AS)
- Bachelor’s degree (e.g., BA, BS)
- Graduate degree (e.g., MA, MS)
- Professional degree (e.g., MD, JD)

**Are you in a committed, romantic relationship?** *(Please check one.)*

- Yes
- No

**What is your current marital status?** *(Please check one.)*

- Single
- Married
- Separated
- Divorced
- Widowed

**What is your current employment activity?** *(Please check ALL that apply.)*

- Working full-time (30 or more hours)
- Working part-time (less than 30 hours)
- Full-time student (12 or more credits)
- Part-time student (less than 12 credits)
- Unemployed
- Retired
- Other: _______________________

**Please fill in names of all HOPE party participants. Answer each question about each person by checking (✓) the box with the best answer or by circling Yes or No**.

| **HOPE Party Participant** Letter / Name: | **1.**  What is your relationship to this person?  *(Check One Answer.)* | | | | | **2.**  Is this person a “friend” on a Website like Facebook, MySpace, or Tagged? |
| --- | --- | --- | --- | --- | --- | --- |
|  | **Family Member** | **Good Friend** | **Romantic Partner (spouse, boyfriend, girlfriend…)** | **Acquain-tance** | **Just met** |  |
| A. | ❑ | ❑ | ❑ | ❑ | ❑ | Yes No |
| B. | ❑ | ❑ | ❑ | ❑ | ❑ | Yes No |
| C. | ❑ | ❑ | ❑ | ❑ | ❑ | Yes No |
| D. | ❑ | ❑ | ❑ | ❑ | ❑ | Yes No |
| E. | ❑ | ❑ | ❑ | ❑ | ❑ | Yes No |
| F. | ❑ | ❑ | ❑ | ❑ | ❑ | Yes No |
| G. | ❑ | ❑ | ❑ | ❑ | ❑ | Yes No |
| H. | ❑ | ❑ | ❑ | ❑ | ❑ | Yes No |
| I. | ❑ | ❑ | ❑ | ❑ | ❑ | Yes No |
| J. | ❑ | ❑ | ❑ | ❑ | ❑ | Yes No |
| K. | ❑ | ❑ | ❑ | ❑ | ❑ | Yes No |
| L. | ❑ | ❑ | ❑ | ❑ | ❑ | Yes No |
| M. | ❑ | ❑ | ❑ | ❑ | ❑ | Yes No |
| N. | ❑ | ❑ | ❑ | ❑ | ❑ | Yes No |
| O. | ❑ | ❑ | ❑ | ❑ | ❑ | Yes No |

| **HOPE Party Participant** Letter / Name: | **3.**  **How close** do you feel to this person?  *(Check One Answer.)* | | | **4.**  Do **you GET information** about HIV/STDs from this person? | **5.**  Do **you GIVE information** about HIV/STDs to this person? |
| --- | --- | --- | --- | --- | --- |
|  | **Do NOT feel close to** | **SOMEWHAT close to** | **VERY close to** |  |  |
| A. | ❑ | ❑ | ❑ | Yes No | Yes No |
| B. | ❑ | ❑ | ❑ | Yes No | Yes No |
| C. | ❑ | ❑ | ❑ | Yes No | Yes No |
| D. | ❑ | ❑ | ❑ | Yes No | Yes No |
| E. | ❑ | ❑ | ❑ | Yes No | Yes No |
| F. | ❑ | ❑ | ❑ | Yes No | Yes No |
| G. | ❑ | ❑ | ❑ | Yes No | Yes No |
| H. | ❑ | ❑ | ❑ | Yes No | Yes No |
| I. | ❑ | ❑ | ❑ | Yes No | Yes No |
| J. | ❑ | ❑ | ❑ | Yes No | Yes No |
| K. | ❑ | ❑ | ❑ | Yes No | Yes No |
| L. | ❑ | ❑ | ❑ | Yes No | Yes No |
| M. | ❑ | ❑ | ❑ | Yes No | Yes No |
| N. | ❑ | ❑ | ❑ | Yes No | Yes No |
| O. | ❑ | ❑ | ❑ | Yes No | Yes No |

FOLLOW UP QUESTIONS

**Since the HOPE party, have you used the HOPE website or other on-line activities.**

- Yes
- No

**If Yes, how did you get on-line? (check all that apply)**

- Computer
- Laptop
- Tablet
- E-reader
- Cell phone
- Other: _______________________

**What HOPE activities did you do on-line? (check all that apply)**

- Visit HOPE website
- Post or blog on the HOPE website
- Tweet on Twitter
- Post on Facebook
- Other: _______________________

**In the last 30 days, how many days have you been on-line to do a HOPE activity? _____________________________**

**If Not, why not? (check all that apply)**

- I do not have computer access
- No time
- Did not want to
- Other: ________________________

**What is the best way for us to contact you to let you know that it is time to complete the survey? (**Please check one)

| - Telephone |
| --- |
| - E-mail |
| - Texting |
| - Facebook private message |
| - US Mail |
| - Other |

**What is the best way for you to complete the survey? (**Please check one)

| - I prefer to do the survey on a computer (webpage on the internet) |
| --- |
| - I prefer to do the survey on my smart phone with a mobile phone format |
| - I prefer to do the survey over the phone |
| - I prefer for you to send the survey to me through the mail |
| - I prefer to come in to the U of M office and do the survey in person |
| - Other: _________________________________________ |

**Thank you very much for your time in completing this survey!**
