# Supplementary material for: Uptake of and Engagement With an Online Sexual Health Intervention (HOPE eIntervention) Among African American Young Adults: Mixed Methods Study
Source: J Med Internet Res. 2021 Jul 16;23(7):e22203. doi: 10.2196/22203 (PMC8325088; doi:10.2196/22203)
Supplement: Multimedia Appendix 6 [file jmir_v23i7e22203_app6.docx]

**Appendix 6.** Extended model of user engagement based on qualitative interview data

|  | Examples (+ Positive/- Negative) |
| --- | --- |
| **Uptake** |  |
| *Individual-Level Characteristics* | |
| Awareness | +“Was there a particular reason why you went to the [HOPE] page?” (INTERVIEWER) “Just to visit… And to see what was on it.” (P008)  - “I've never heard of [the Website].” (P018) |
| Motivation | *Referral to HOPE Website*  + “I do Big Brother/Big Sister so I do a lot of public speaking to them, like I go to schools and stuff. So it be more like 13 year olds, 14 year olds like people that notice, they're just starting to get sexually active….I refer people…so they can get more information….Like maybe if you know how many diseases actually are out here... you won't be just trying to just go ahead and have sex anytime as you can.” (P010)  *Providing Information Learned on HOPE Online*  + “What makes you decide to visit? Do you have a particular question in mind?” (INTERVIEWER) “It just depends on what's going on in my day to day life. Sometimes I go in there to see, like can I help people I know with questions they have or, if I can direct them in the right direction for certain things like that.” (P012) |
|  | *Providing Information Learned on HOPE Online*  + “What makes you decide to visit? Do you have a particular question in mind?” (INTERVIEWER) “It just depends on what's going on in my day to day life. Sometimes I go in there to see, like can I help people I know with questions they have or, if I can direct them in the right direction for certain things like that.” (P012) |
| *Necessary Conditions* | |
| Technology Access | +/- “I generally use my cell phone, for internet...I just get in on there every day. Facebook...I don't have no other website. I know I'll be Googling it and that's about it.” (P008)  - “[The Internet]’s at my granny house and that’s it.” (P009) How often do you get on the Internet from your grandmother's place? (INTERVIEWER) “Not that often.” (P009) |
| Perceived Time | - “I don't need it for me to be messing with it. And I don't really got time to be on the internet. I'm not interested. I might shop online sometimes, but that's probably about all, and somebody do it for me.” (P002) |
| Trust — Institutional | *Churches*  + (INTERVIEWER) “Who did you go to the party with?” “Some of my church friends.” (P018) |
|  | *YOUR Center*  + “If you did not have that website, are there any other websites you would try to go to for that same kind of information?” (INTERVIEWER) “I never really try a different website, but I actually have a resource, a person that I can go to, and she'd be a friend…She actually works at YOUR Center.” (P004) |
| Trust — Technological | *Credible Information*  + “You have to be really careful with where you get your information from on the internet, a trusted site. (INTERVIEWER) And you trust Hope online?  “Yeah, definitely.” (P002) |
|  | *Technology-facilitated privacy breaches*  - “... You can just go through somebody's computer and lookin' at those stuff they might just take it and put it on their Facebook page, or put it on their Twitter, or screenshot it and put it on their Instagram, tag other people in it so other people can see it.” (P010) |
|  | *Fighting*  - “I don’t like using technology, cause things be happening that I don’t want. That’s why I don’t like being on the Internet too much... I don’t argue, that’s the key. I don’t argue on Facebook…” (P017) |
|  | *Gossip*  - “I heard…people would…try to blast out all the gay people…and then I heard about…a page where they were basically saying all the people that had AIDS…I believe that everybody's personal business is their business.”  (P004) |
| **Initial Engagement** | |
| *Intervention Attributes* | |
| Aesthetics/Sensory Appeal | + “It's definitely very appealing, the colors and it shows you different things just from first glance about different things people are into sexually…I like the fact that it was very inviting. You login and it actually keeps your interest and wasn't just black and white and whole bunch of a... You know, when you see a lot of words…” (P003) |
| Challenge/Ease of Use | *Easy to Understand*  + “…when you look up stuff on Google and different things, you find a whole bunch of stuff, but on the HOPE website it basically is like a general what this is, what you can do to prevent it, or if you have it what you can do to get help about it which people like…everything is answered in one little brief paragraph…. That's what people want.” (P011) |
|  | *Some Challenge*  + “I actually think it's great because it gives a lot of information that people might not know….they just guess or assume things, so it's very great for people who don't know about STDs….And it's actually pretty good for people who do know, because you can always learn new stuff off of the website, 'cause I have.” (P012) |
| **Engagement** |  |
| Novelty | - “I went to get more information for our presentation and stuff. It's kinda... It's been a little minute since I've actually went and used it, but I go just to glance sometimes, to see what goes on. I know before it wasn't quite updated completely the last time I went on there. But this, seeing what HOPE is and under it, it had the definition right there and different activities you can do. So it was mainly just going on there to get more information about their definition of it and things like that.” (P005) |
| Interactivity | + “I did come across some things I didn't know. People were very active on there as far as discussing things, giving information. So I thought that was pretty cool.” (P012)  - “So they can post pictures or have a profile and have people follow them or they can follow other people…maybe they're going through the same thing and they wanna follow those sort of people.” (P001) |
| **Intervention Context** | |
| Promotion of Intervention | *Communication*  - “Is there anything that you can think of that might encourage young people in your community to go to websites like that?” (INTERVIEWER) “It takes people who know about the HOPE parties…to really get it out there.” (P007) |
|  | *Contests on Social Media*  -“Were you interested in the money?” (INTERVIEWER)” Yeah.” (P001) |
| Platform of Intervention | *Website*  -“It's very great for people who don't know about STDs and stuff like that. And it's actually pretty good for people who do know, because you can always learn new stuff off of the website, 'cause I have.” (P003) |
|  | *Social Media*  -“I wouldn't recommend anyone put anything personal about themselves on any type of social media.” (P012) |
