# Supplementary material for: Uptake of and Engagement With an Online Sexual Health Intervention (HOPE eIntervention) Among African American Young Adults: Mixed Methods Study
Source: J Med Internet Res. 2021 Jul 16;23(7):e22203. doi: 10.2196/22203 (PMC8325088; doi:10.2196/22203)
Supplement: Multimedia Appendix 7 [file jmir_v23i7e22203_app7.docx]

Appendix 7. Empirical support for each element of the proposed model

| MODEL COMPONENT | Rationale/Support |
| --- | --- |
| Individual Characteristics - Education | Education is a factor from the Diffusion of Innovation (DOI) theory. It is included in the model due to results from the individual statistical model indicating a marginally significant relationship between education and uptake. The line is dotted because the relationship was only marginally significant. |
| Individual Characteristics - Motivation | Motivation comes from the O’Brien and Toms model of Engagement. It has supporting evidence from the interviews in regards to motivation to use the eIntervention being rooted in referring others to the intervention and sharing information learned from the intervention with others (see the rows with these names in the former Table 4, now Table 5). The solid line indicates a relationship with uptake. |
| Individual Characteristics - Awareness | Awareness comes from the O’Brien and Toms model of Engagement. It has supporting evidence from the interviews in which participants said they did not know about the eIntervention (see the rows with these names in the former Table 4, now Table 5). The solid line indicates a relationship with uptake as well as being influenced by the promotion of the intervention, which interview participants indicated was necessary to make them aware of it. |
| Necessary Conditions - Technology Access | Necessary conditions are facts that must be true for intervention uptake; however, they are insufficient on their own to promote uptake. Technology Access was supported by the survey results in which these were the top 2 reasons given for not using the HOPE eIntervention in the past 30 days (was Table 2, now Table 3) and the interview results which further supported this point (see rows with these labels the former Table 4, now Table 5). The solid line indicates a “necessary condition” relationship with uptake. |
| Necessary Conditions - Perceived Time | Perceived time was supported by the survey results in which these were the top 2 reasons given for not using the HOPE eIntervention in the past 30 days (was Table 2, now Table 3) and the interview results in which participants spoke of how business in terms of jobs or parenting made it difficult to find time to use the eIntervention (see rows with these labels the former Table 4, now Table 5) The solid line indicates a “necessary condition” relationship with uptake. |
| Necessary Conditions - Trust | Institutional and Technological trust originate from the Trust-centered design model that was used to develop the intervention, and was supported by interview results (see rows with these labels the former Table 4, now Table 5). The solid line indicates a “necessary condition” relationship with uptake. |
| Intervention Context - Social Influence (Gender Homophily, prior relationships) | Social influence originates from the DOI theory; it indicates that homophily impacts innovation diffusion, such that one is more likely to take up interventions used by those who are socially similar to oneself. The presence of gender homophily in the model was supported by the party-level statistical analyses in which this variable was a marginally significant predictor of uptake. The line is dotted because the relationship was marginally significant. |
| Intervention Context - Promotion of Intervention (communication, contests) | Promotion of the intervention came from the qualitative results in which several participants said that more communications about the website were needed and three interview participants indicated that contests had affected their re-engagement. This is depicted as influencing re-engagement, as this was the impact of the social (the line is dotted because the relationship was supported by data from only three participants). |
| Intervention Context - Platform of intervention (website, social media) | Inclusion of the platform of the intervention was supported by interview results as platform influenced trust, specifically regarding use of social media to discuss sexuality (see rows with the relevant labels the former Table 4, now Table 5) The solid line indicates a relationship with trust. |
| Intervention Attributes - Aesthetics/Sensory appeal, Challenge/ease of use, Novelty, Interactivity | There four intervention attributes (aesthetics/sensory appeal, challenge/ease of use, novelty, and interactivity) originated from O’Brien and Toms Model of Engagement and was supported by interview data (see the rows with these names in the former Table 4, now Table 5) The different attributes influence initial engagement, engagement, and disengagement as indicated by the solid lines. |
| Uptake | We added in uptake as a novel addition prior to engagement as we found through the study that initial contact with the intervention could not be assumed, and was a necessary precursor to interaction with the intervention. |
| Initial engagement, Engagement, Disengagement, Re-Engagement | These constructs came from the O’Brien and Toms model. Supporting evidence is in the interview data, where influencers in the Figure are located under each relevant phase in the Table (see rows with the relevant labels the former Table 4, now Table 5). |
